# Eales' Disease: When the Rare Sounds Frequent

**DOI:** 10.1155/2021/1056659

**Published:** 2021-08-09

**Authors:** Beatriz Oliveira Lopes, Margarida Sena Brízido, Ana Isabel Reis, Margarida Maria Miranda, Susana Morais Pina

**Affiliations:** ^1^Department of Ophthalmology, Beatriz Ângelo Hospital, Loures, Portugal; ^2^Department of Medicine, Beatriz Ângelo Hospital, Loures, Portugal

## Abstract

Eales' disease is a peripheral occlusive retinal phlebitis, with an unclear pathogenesis. The classic association with hypersensitivity to *Mycobacterium tuberculosis* protein infers that immunologic disturbance may be involved. Here, we described three cases of Eales' disease. All patients are Caucasian men aged 27-58 years and presented with vitreous hemorrhage and/or peripheral venous vasculitis. Tuberculin skin sensitive test (*Mantoux* screening test) and interferon-gamma release assay (IGRA) were positive in all patients. Therapeutic approach included antituberculosis therapy and systemic steroids, associated or not to immunosuppressive therapy, and retinal scatter photocoagulation in all cases. Antivascular endothelial grow factor (*VEGF*) intravitreal injections were also required in two cases. Since various retinal diseases can resemble this presentation, Eales' disease is considered a diagnosis of exclusion. Early diagnosis and appropriate therapeutic approach are both essential to accomplish disease control and reduce ophthalmologic complications.

## 1. Introduction

Eales' disease is a primary idiopathic peripheral obstructive retinal vasculopathy, reported in 1880 by Henry Eales for the first time [1]. A relation to ocular inflammation was described few years later [2, 3]. Healthy young men are the most affected by this disease, which is more prevalent in India and the Middle East [1]. In recent years, more cases affecting older adults have been described.

The clinical findings of Eales' disease include perivascular phlebitis and secondary proliferative retinopathy with neovascularization, recurrent vitreous hemorrhages, and possible tractional retinal detachment [1, 2]. Other signs of ocular inflammation as vitritis and anterior chamber cell reaction may also be present. The clinical course of Eales' disease is highly variable, presenting itself with gradual progression in few cases and temporary or even permanent remission in others [4].

Although the pathophysiology is poorly clarified, literature suggests that there is a clear association between Eales disease and *Mycobacterium tuberculosis* [2, 4, 5].

Different therapeutic approaches are suggested for Eales' disease, depending on its clinical stage [3, 5]. The typical retinal vasculitis usually requires treatment with systemic steroids in combination with complete antituberculosis therapy for a minimal period of 9 months. Steroid- paring immunosuppressive agents, like methotrexate, cyclosporine, and azathioprine, are usually reserved for cases which corticosteroids are not sufficient, need to be discontinued, or are contraindicated. Peripheral retinal scatter photocoagulation and anti-*VEGF* intravitreal injections are also essential therapeutic approaches in cases of proliferative disease and important retinal ischemia [2, 5]. In case of secondary ocular complications such as tractional retinal detachment and/or persistent vitreous hemorrhage, pars plana vitrectomy surgery may improve visual prognosis [2].

## 2. Clinical Cases

### 2.1. Patient 1

**Figure 1:**
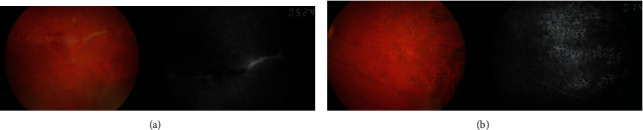
Retinography and fluorescein angiography evidencing (a) active venous vasculitis with perivascular hemorrhages and retinal ischemia in the nasal quadrants OD in patient 1and (b) after medical treatment and scatter laser photocoagulation.

A healthy 27-year-old Caucasian male presented with progressive blurred vision in the right eye (OD) (Table 1). He denied ocular pain, headaches, and other ocular or systemic symptoms. Best corrected visual acuity (BCVA) was 10/10 in both eyes. Anterior segment evaluation showed low-grade iridocyclitis in the OD. Dilated ocular fundus examination revealed moderate vitritis, optic disc edema, vascular tortuosity, and venous vasculitis with perivascular hemorrhages mainly in the nasal quadrants of the OD (Figure 1). Fluorescein angiography showed severe venous vasculitis with important retinal ischemia in the correspondent quadrants of the same eye (Figure 1 and Table 1). Unilateral panuveitis with retinal vasculitis, prompted for the search of an etiology study while topical therapy with prednisolone and bromofenac, was implemented.

Initial investigation included hemogram, platelet count, hepatic and renal function, erythrocyte sedimentation rate, human leukocyte antigen (HLA) B51, antinuclear antibody, lysozyme, angiotensin-converting enzyme (ACE), and infectious serologic analysis. No suspicious lesions indicating active tuberculosis or sarcoidosis were found in the computed tomography (CT) of the thorax; although, it showed a cisural micronodule with a 4 mm ganglion (Table 1). A further systematic review was unremarkable. Tuberculin skin sensitive test and *IGRA test* (5.59) were positive, and Eales' disease was considered the likely diagnosis.

Besides topical therapy, corticoid therapy (1 mg/Kg/day oral prednisolone) with slow progressive withdrawal was started, associated with quadruple antituberculosis therapy (ethambutol, rifampicin, pyrazinamide and isoniazid). Oral methotrexate (15 mg/week) was introduced two months later, keeping slow progressive corticosteroids withdrawal with clinical improvement (Table 1). Sectorial laser photocoagulation of the ischemic nasal quadrants of the retina was performed after vasculitis remission (Figure 1).

Two years after diagnosis, having completed one year of antitubercular and two years of immunosuppressive treatment, the patient remains clinically stable, with no signs of active vasculitis and no need for further therapy.

### 2.2. Patient 2

**Figure 2:**
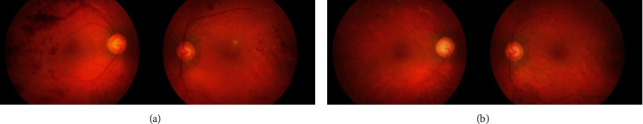
Image of ocular fundus showing (a) active venous vasculitis with hemorrhages in all retinal quadrants in both eyes in patient 2 and (b) after treatment, including scatter laser photocoagulation and intravitreal anti-VEGF therapy.

**Figure 3:**
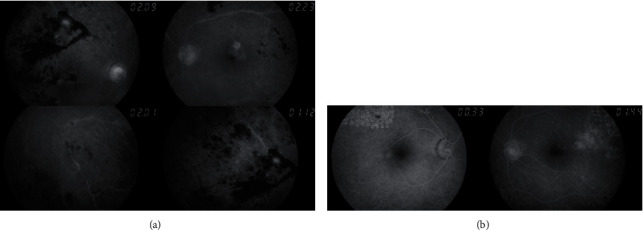
Fluorescein angiography showing (a) retinal venous vasculitis, perivascular hemorrhages, and severe retinal ischemia in both eyes in patient 2 and (b) after treatment, including scatter laser photocoagulation.

A healthy 48-year-old Caucasian male came to the emergency room with acute bilateral decrease visual acuity and floaters (Table 1). He denied ocular pain, fever, anorexia, and other systemic symptoms. BCVA in OD was 6/10 and in OS was 5/10. Biomicroscopic examination was unremarkable in both eyes. Dilated ocular fundus examination showed vitritis, vitreous haemorrhage, and extensive retinal venous vasculitis with haemorrhages in all retinal quadrants and macular edema (Figure 2). Fluorescein angiography showed bilateral severe occlusive venous vasculitis associated with paramacular, midperipheral, and peripheral retinal ischemia (Figure 3), and spectral domain optical coherence tomography (*SD-OCT*) revealed cystoid macular edema (CME) in both eyes (Table 1).

Investigation of the etiology included hemogram, inflammatory markers (C-reactive protein and erythrocyte sedimentation rate), human leukocyte antigen (HLA) B51, lysozyme, angiotensin-converting enzyme (ACE), infectious serologic testing and hemoglobin electrophoresis, coagulation, and prothrombotic evaluation that were all unremarkable, except for tuberculin skin sensitive test (20 mm) and IGRA (>10) which were positive. X-ray and CT scan of the thorax also revealed unspecific small bilateral hilar ganglia (Table 1).

Eales' disease was suspected, and patient started complete quadruple antituberculosis treatment, which was maintained for nine months, associated with oral corticoid therapy (prednisolone 1 mg/Kg/day) with slow progressive withdrawal and subsequent introduction of oral methotrexate (20 mg/week) in order to achieve control of the disease. Serial treatment regime with anti-*VEGF* vitreous injections was also performed in both eyes (in a total of 11 in each eye). Laser photocoagulation was also required in both eyes over four sessions (Figure 2 and Table 1). This therapeutic approach allowed progressive clinical improvement.

After two years of follow-up, the patient maintains immunosuppression with oral methotrexate (20 mg/week) associated with prednisolone (2.5 mg/day) and achieved almost complete visual acuity recovery for 10/10 in both eyes, although with significant restriction of visual field, induced by both severe retinal ischemia and photocoagulation (Table 1).

### 2.3. Patient 3

**Figure 4:**
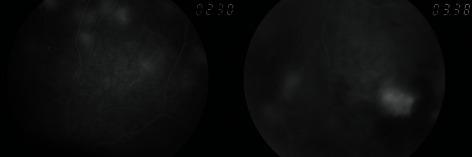
Fluorescein angiography showing significant occlusive vasculitis associated with peripheral ischemia, hemorrhages, and neovascularization in both eyes in patient 3.

A 58-year-old Caucasian male with known medical history of dyslipidemia, diabetes mellitus type 2 for 15 years, and ischemic stroke 15 years before presented to the emergency room with complaints of floaters in his left eye over the previous 6 months (Table 1). At presentation, BCVA was 8/10 OD and 5/10 OS. Anterior segment evaluation was unremarkable. Fundoscopy revealed peripheral retinal hemorrhages in both eyes and irregular macular reflex, inferior vitreous hemorrhage, vascular remodeling, and neovascularization in OS (Table 1). Fluorescein angiography showed retinal venous vasculitis with peripheral ischemia and neovascularization in both eyes, with no suggestive appearance of diabetic retinopathy (Figure 4). Patient also performed *SD-OCT* which revealed epiretinal membrane (ERM) with CME in the OS (Table 1).

Detailed clinical history and thorough systemic examination including serologic analysis, hemogram, inflammatory markers, protein, and hemoglobin electrophoresis were unremarkable. X-ray and CT scan of the thorax evidenced pulmonary parenchyma with apical reticular pattern and residual fibrotic and retracted changes (Table 1). Once other causes of vasculitis and infections have been ruled out and tuberculin skin sensitive test (41 mm) and *IGRA* (2.77) were positive, Eales' disease was considered.

Patient started a complete quadruple antituberculosis treatment associated with oral corticoid therapy (prednisolone 1 mg/Kg/day) with progressive withdrawal. Scatter laser photocoagulation and three anti-VEGF vitreous injections were also performed in both eyes (Table 1). Although poor patient's compliance, clinical improvement with complete regression of vasculitis and neovascularization was achieved.

Almost 4 years after diagnosis, patient remains clinically stable and had significant visual acuity recovery (BCVA was 10/10 OD and 8/10 OS), maintaining close follow-up and no need for treatment.

## 3. Discussion

Defined as a primary idiopathic disorder of peripheral retinal vessel, Eales' disease pathophysiology is still poorly understood. This disease classically progresses in sequential stages involving retinal inflammation, occlusion, and neovascularization [2, 5]. Early inflammatory stage presents with retinal periphlebitis, which manifests as sheathing of blood vessels, primarily venous. This vasculitis leads to capillary occlusion in the middle stage which contributes to retinal ischemia with increased vascular endothelial growth factor [2]. Neovascularization seen at the boundaries between perfused and nonperfused zones of the retina is observed consequently in the late proliferative stage. It can happen up to 80% of the cases [1, 2], unlike disc neovascularization, which is rare [2]. Recently, it has been created a new 4 stage-grading classification in order to facilitate assess severity and functional prognosis of the disease [1, 2] (Table 2).

Eales is a bilateral condition in approximately 90% of patients. However, due to the inherent asymmetry, it can manifest itself unilaterally, as observed in patient 1, as sudden blurring of vision or floaters, caused by vitreous hemorrhage [1]. Sometimes, this presentation may difficult the correct diagnosis. In our case series, neovascularization associated with vitreous hemorrhages was present in patients 2 and 3. We speculated that in the first patient, the diagnosis was made sooner at an earlier stage, which allowed preventing this serious complication.

Immunologic mechanisms, mainly cell-mediated, have been proposed for Eales' etiology [3, 5]. From the various causes suggested, *Mycobacterium tuberculosis* is the most common reported. Although the role of this antigen is not completely determined, several studies propose that Eales disease is strongly related to both tuberculin protein hypersensitivity and previous exposure to tuberculosis [2, 5]. Indeed, the *Mycobacterium tuberculosis* genome had been detected through polymerase chain reaction studies in epiretinal membranes and vitreous samples of several Eales patients with tuberculin skin test positivity [6, 7].

In our case series, tuberculin skin sensitive test as well as *IGRA* test was positive in all patients, supporting the possible etiologic role of *Mycobacterium tuberculosis* in the pathogenesis of this entity and contributing to the diagnosis as well. However, the diagnosis of Eales' disease requires exclusion of several other ocular or systemic inflammatory and noninflammatory diseases which can easily resemble its features [5]. Branch retinal vein occlusion, proliferative diabetic retinopathy, Coats disease, and sickle cell disease are all retinal entities which manifest with similar peripheral retinal nonperfusion that must be ruled out [1, 5]. It is also important to exclude systemic infections and noninfectious diseases such as syphilis, sarcoidosis, toxoplasmosis, systemic lupus erythematosus, or Behçet disease [5, 8]. As we reported with our case series a detailed clinical history, ocular and systemic examination with appropriate diagnostic testing, including serologic analysis, hemoglobin electrophoresis, tuberculin skin sensitive test, and/or *IGRA* is usually required and essential for a complete diagnosis approach.

Treatment approach is defined in accordance with the clinical stage of the disease. Systemic corticosteroids are the mainstay of treatment for active vasculitis, especially in severe bilateral cases [2]. Although the role of antituberculosis therapy in Eales disease remains some point controversial, authors believe that its association with corticosteroids is extremely important in the disease control when IGRA and/or tuberculin sensitivity test are positive, or patients present with a presumably lung tubercular lesion [2, 8, 9]. In this way, all presented cases have been treated with the association of systemic corticosteroids and antituberculosis therapy, and two of them required immunosuppressive therapy with oral methotrexate as well, to achieve disease control and to prevent the adverse effects of corticosteroid treatment complications. In the proliferative stage, retinal photocoagulation combined with anti-*VEGF* intravitreal injections is the treatment of choice [10]. In cases of macular edema, anti-*VEGF* intravitreal injections are also indicated [9, 11]. Retinal photocoagulation was performed in all cases, and anti-*VEGF* intravitreal injections required in patients 2 and 3 for macular edema and/or associated neovascularization treatment. Photocoagulation is not recommended in case of active vasculitis, as it may release more angiogenic factors, with potential worsening of neovascularization [5]. Previous treatment with corticosteroid or immunosuppressive therapy may avoid demand of subsequent photocoagulation [5]. The major indication for pars plana vitrectomy is persistent vitreous hemorrhage and less commonly, tractional retinal detachment or ERM [5, 12]. Patient 3 developed ERM, although with no need for surgical treatment yet.

Although the progressive nature of this disease and the possible associated ocular complications such as tractional retinal detachment, neovascular glaucoma, rubeosis iridis, and cataract, visual prognosis is generally good. Recurrent vitreous hemorrhages are the most frequent cause of visual loss, which is transitory most of the time, as observed in our patients. Therefore, patients with Eales' disease can have a hopeful prognosis, being mainly affected by early diagnosis, prompt treatment, and regular long-term follow-up.

Our case series intend to draw attention to this rare entity which has become more frequent in the western world and between older adults. In this way, clinical suspicious and knowledge of the appropriate therapeutic management of the disease are essential to prevent clinical progression and the onset of ocular complications, as we accomplished with our patients.

## Figures and Tables

**Table 1:** Description of the presented cases. Abbreviations: OD: right eye; OS: left eye; FA: fluorescein angiography; *SD-OCT*: spectral domain optical coherence tomography; ERM: epiretinal membrane; CME: cystoid macular edema; CT: computed tomography; IGRA: interferon-gamma release assay; VEGF: antivascular endothelial grow factor.

	Case 1	Case 2	Case 3
Sex	Male	Male	Male
Age (years)	27	49	58
Laterality	Unilateral	Bilateral	Bilateral
Clinical presentation	Progressive blurred vision in the right eye	Acute bilateral decrease visual acuity and floaters	Floaters in the left eye over the previous 6 months
Anterior segment evaluation	Low-grade iridocyclitis	Unremarkable	Unremarkable
Fundus features	Vitritis, optic disc edema, venous vasculitis, and perivascular haemorrhages in nasal quadrants (OD)	Bilateral vitritis, vitreous hemorrhage, retinal vasculitis, hemorrhages in all retinal quadrants, and macular edema	Bilateral peripheral hemorrhages, vascular remodeling, neovascularization, vitreous hemorrhage, and macular edema (OS)
Ophthalmology multimodal evaluation	FA: venous vasculitis with retinal ischemia	FA: bilateral occlusive venous vasculitis with retinal ischemia SD-OCT: bilateral CME	FA: bilateral venous vasculitis with peripheral ischemia, neovascularization *SD-OCT*: ERM+ CME (OS)
Tuberculin skin sensitive and *IGRA* test	Positive	Positive	Positive
Thorax TC	Cisural micronodule with a 4 mm ganglion	Unspecific small bilateral hilar ganglia	Apical reticular pattern, residual fibrotic, and retracted changes
Systemic evaluation	Negative	Negative	Negative
Systemic treatment	Quadruple antitubercular treatment, prednisolone (1 mg/kg/day) with progressive withdrawal, and methotrexate (20 mg/week)	Quadruple antitubercular treatment, prednisolone (1 mg/kg/day) with progressive withdrawal, and methotrexate (20 mg/week)	Quadruple antitubercular treatment and prednisolone (1 mg/kg/day) with progressive withdrawal
Ocular treatment	Topical prednisolone and bromofenac Sectorial laser photocoagulation	Scatter laser photocoagulation and intravitreal anti-*VEGF* in both eyes	Scatter laser photocoagulation and intravitreal anti-VEGF in both eyes
Time on follow-up	2 years	2 years	4 years
Follow-up	Clinically stable with no need for therapy Complete visual acuity recovery	Clinically stable, under methotrexate (20 mg/week) and prednisolone (2.5 mg/day) Significant visual acuity recovery, restriction of visual field	Clinically stable with no need for therapy Significant visual acuity recovery

**Table 2:** Disease severity classification system [13].

Stage	Description
Ia	Periphlebitis of small caliber vessels with superficial retinal hemorrhages
Ib	Periphlebitis of large caliber vessels with superficial retinal hemorrhages
IIa	Capillary nonperfusion
IIb	Neovascularization elsewhere/of the disc
IIIa	Fibrovascular proliferation
IIIb	Vitreous hemorrhage
IVa	Traction/rhegmatogenous retinal detachment
IVb	Rubeosis iridis, neovascular glaucoma, complicated cataratac, and optic atrophy

## Data Availability

Data are available within the article.
